# Classification of Stiff-Knee Gait Kinematic Severity after Stroke Using Retrospective k-Means Clustering Algorithm

**DOI:** 10.3390/jcm11216270

**Published:** 2022-10-25

**Authors:** Frédéric Chantraine, Céline Schreiber, José Alexandre Carvalho Pereira, Jérôme Kaps, Frédéric Dierick

**Affiliations:** 1 Laboratoire d’Analyse du Mouvement et de la Posture (LAMP), Centre National de Rééducation Fonctionnelle et de Réadaptation—Rehazenter, Rue André Vésale 1, 2674 Luxembourg, Luxembourg; 2 Medical Department, Centre National de Rééducation Fonctionnelle et de Réadaptation—Rehazenter, Rue André Vésale 1, 2674 Luxembourg, Luxembourg; 3 Physiotherapy Department, Centre National de Rééducation Fonctionnelle et de Réadaptation—Rehazenter, Rue André Vésale 1, 2674 Luxembourg, Luxembourg; 4 Faculté des Sciences de la Motricité, UCLouvain, Place Pierre de Coubertin 1-2, 1348 Ottignies-Louvain-la-Neuve, Belgium

**Keywords:** clusters, kinematics, diagnostic, locomotion, hemiparetic

## Abstract

Nowadays, a classification system for unilateral stiff-knee gait (SKG) kinematic severity in hemiparetic adult patients after stroke does not exist. However, such classification would be useful to the clinicians. We proposed the use of the k-means method in order to define unilateral SKG severity clusters in hemiparetic adults after stroke. A retrospective k-means cluster analysis was applied to five selected knee kinematic parameters collected during gait in 96 hemiparetic adults and 19 healthy adults from our clinical gait analysis database. A total of five discrete knee kinematic clusters were determined. Three clusters of SKG were identified, based on which a three-level severity classification was defined: unbend-knee gait, braked-knee gait, and frozen-limb gait. Preliminary construct validity of the classification was obtained. All selected knee kinematic parameters defining the five clusters and the majority of usual kinematic parameters of the lower limbs showed statistically significant differences between the different clusters. We recommend diagnosing SKG for values strictly below 40° of knee flexion during the swing phase. Clinicians and researchers are now able to specify the level of kinematic severity of SKG in order to optimize treatment choices and future clinical trial eligibility criteria.

## 1. Introduction

Regardless of whether being in the subacute or chronic phase, adult patients presenting an upper motor neuron lesion, such as stroke or traumatic brain injury, often exhibit sensorimotor lower limb deficits on the paretic side, that can vary widely in severity [1,2]. Among these deficits, stiff-knee gait (SKG) or stiff-legged gait [3,4,5,6] is one of the most commonly observed gait disorders [4,5], and affects approximately 60% of stroke patients with gait disorders [7]. However, the SKG definition varies somewhat in the literature [4,8,9], which does not facilitate its application in routine clinical practice. From a functional point of view, restricted knee flexion may affect gait stability due to insufficient foot clearance [10], thereby increasing the risk of falls. In addition, compensatory movements are performed to clear and move the foot forward during the swing phase, such as homolateral hip hiking or abduction, or contralateral vaulting [10,11].

Unfortunately, the physiopathology of SKG is not fully understood, and several hypothetical mechanisms involving the knee, ankle, or hip joints have been proposed. Firstly, SKG could be related to an increased stretch reflex activity of the *quadriceps femoris* muscle, particularly the *rectus femoris* head, during the end of the stance phase and the first part of the swing phase [3,11,12,13]. Secondly, it could be related to a lack of ankle push-off power at the end of the stance phase due to weakness of the *triceps surae* muscle [8], and, thirdly, to a hip flexor muscle weakness that could alter the hip pull-off at toe-off. All these three mechanisms isolated or in combination decrease the passive knee flexion angle during the swing phase [14]. Recently, a method to distinguish the main cause of SKG between the lack of ankle push-off, *quadriceps femoris* braking activity, and lack of hip pull-off has also been proposed by analyzing the temporal occurrence of peaks of malleolus vertical acceleration, knee flexion acceleration, and hip flexion acceleration during the preswing phase [8].

Previous studies have included SKG patients based on visual observation during a clinical examination [12,15,16] or different quantitative criteria [4,8,9,17,18] of a reduction in peak knee flexion kinematics. Goldberg et al. [9] even proposed an index to classify cerebral palsy patients into SKG and non-SKG, based on four knee joint kinematics parameters, but without considering severity and assuming that some cases could not be classified and were defined, in this case, as borderline.

In patients with upper motor neuron injury, a quantitative metric of unilateral SKG severity is still lacking [19] and no specific classification has yet been developed, perhaps due to the lack of sufficiently large samples of patients in whom quantitative gait analysis has been performed and to the complexity of classifying patients with very heterogeneous knee gait patterns [7]. In clinical practice, sagittal plane knee excursion may be almost (<10°) or completely absent in some post-stroke patients throughout the swing phase [3,20], while a group of less impaired subjects may maintain some knee mobility and achieve a mean peak flexion angle of about 20 to 40° [4,5,8,20,21,22]. Specific kinematic curves of the knee joint showing a “double bump” during the swing phase, i.e., two flexion angle peaks separated by a distinct extension movement, can also be found in some stroke patients [15,18] and may interfere with the interpretation of visual observation made by clinicians. Therefore, an SKG classification severity is required to allow clinicians to describe knee kinematics more accurately [23]. This classification could establish the framework for selecting therapeutic options and for better design of future clinical trials, which have so far included patients with heterogeneous peak knee flexion angles between 0° and 40–45° during swing phase [4,8,20].

Statistical analysis and, in particular, unsupervised machine learning algorithms such as the k-means method are powerful in classifying gait profiles to orient therapeutic decisions [24]. For example, clustering has been used in many studies to classify gait patterns in children with diplegic cerebral palsy [25,26,27] or flatfeet [28], and in adults with diabetes [29], hip osteoarthritis [30], Alzheimer’s disease [31], or stroke [32,33,34,35]. For the latter group, most studies focused on ankle kinematics. Two studies [32,35] investigated equinus foot deformity as a cause of gait impairment in post-stroke patients. Both studies identified clusters with varying degrees of ankle dysfunction, but no significant differences at the knee level. In another study [33], post-stroke patients were divided into three clusters differentiated by foot position at first ground contact. The first study using non-hierarchical cluster analysis to classify gait patterns in post-stroke patients [34] included several parameters of knee kinematics, but only the peak angle of knee extension in mid-stance was identified as a determinant of cluster assignment.

The purpose of this study was to perform a retrospective unsupervised cluster analysis using the k-means method in adult post-stroke patients, including a limited number of selected sagittal knee kinematics parameters reflecting SKG, in order to identify different severity levels with clinical significance between the proposed SKG clusters. To determine the construct validity of the classification, cluster results were compared to SKG detection criteria and usual gait kinematic parameters were assessed in the different clusters to estimate statistically significant differences between them.

## 2. Materials and Methods

### 2.1. Participants

Ninety-six adult hemiparetic patients and nineteen healthy adults were included in the study. The demographic characteristics of the participants are reported at the beginning of Table 1.

Patients walked at their spontaneous velocity (Table 1) and were retrospectively included from our clinical gait analysis database. They were examined between October 2013 and August 2021 at the Movement Analysis and Posture Laboratory of the Centre National de Rééducation Fonctionnelle et de Réadaptation—Rehazenter (CNRFR—Rehazenter) in Luxembourg, as part of their medical supervised rehabilitation program. Healthy adults walked at a similar velocity as the patients (Table 1) and were collected during a previous study [36].

Inclusion criteria for patients were: left or right hemiparesis due to an ischemic or hemorrhagic stroke and the ability to walk at least 10 m with the help of a cane/crutch or, if necessary, with minimal help by a third person, but without other walking aids (walker, ankle foot orthosis, functional electrical stimulation). Exclusion criteria were designed to be minimal in order to obtain a representative study sample of all kinematic severity presentations of unilateral SKG patients presenting an upper motor neuron lesion and facilitating a posteriori generalizability of the results beyond our sample to transfer it to the real-world patient population [37]. Thus, we excluded only participants who suffered from: rheumatic, metabolic, vascular, or other neurological conditions that could affect gait, Botulinum toxin injections of the lower limbs in the previous 6 months, and neurological or orthopedic surgery of the lower limbs related to the post-stroke condition.

All methods were carried out in accordance with relevant guidelines and regulations. The study was conducted according to the guidelines of the Declaration of Helsinki and approved by the joint ethics committee of the CNRFR—Rehazenter and Hôpital Intercommunal de Steinfort (nr: 202109/01, date of approval: 3 September 2021). Patient consent was waived due to the retrospective nature and statistical purpose of the study. According to the General Data Protection Regulation and the Free Flow Data Regulation (GDPR) in the European Union, no specific consent is needed for statistical results of aggregated data, as it relates to no specific, natural person (GDPR: recital 162) and provided appropriate safeguards are implemented (GDPR: recital 157 and article 89). More, these statistical results may be used for scientific research purpose (GDPR: recital 162). For this study, to ensure the protection of personal data, the raw data were first extracted by one of the authors (C.S.) from a computerized patient record and clinical gait analysis database, and the patients’ identifiers, including family and given names, dates of birth, social security and medical record numbers, addresses, and phone numbers, were de-identified.

### 2.2. Gait Analysis

After a clinical examination to measure the total passive range of motion of the hip, knee, and ankle joints, muscle strength, and stretch reflex activity conducted by the same experienced physiatrist (F.C.), a clinical gait analysis was performed. In order to remain focused on the purpose of the study, only three clinical characteristics that could be directly related to SKG were kept: the stretch reflex activity of the *quadriceps femoris* muscle and the muscle strength of the hip and ankle joints. The stretch reflex activity was assessed in prone position during fast passive knee flexion and scored using the modified Ashworth scale (mAS_4ceps_) [38], and 1+ scores were coded as 1. Hip flexor (MRC_hip_) and ankle plantar flexor (MRC_ankle_) muscles’ strength were assessed using the Medical Research Council (MRC) grading system [39]. The clinical characteristics of the patients are presented in the middle of Table 1.

A subset of 24 retroreflective cutaneous markers was placed on the lower limbs of the patient [36] by anatomical palpation following the guidelines provided by [40] and remaining unchanged over the years. The marker set used allows the application of the biomechanical model proposed by [41]. This model follows the recommendations of the International Society of Biomechanics (ISB) for the definitions of joint coordinate systems and joint centers [42].

The patient then walked barefoot at a self-selected velocity along a 10 m walkway. Ten optoelectronic cameras (OQUS4, Qualisys, Goteborg, Sweden) with a sampling frequency of 100 Hz, and two video cameras (OQUS-2c, Qualisys, Goteborg, Sweden) in the frontal and sagittal planes simultaneously recorded the gait.

At least 6 walking trials were performed, and 5 gait cycles were kept for each participant for averaging. A total of 480 gait cycles were analyzed for the patients and 95 for the healthy subjects. All foot-strike and foot-off events were manually detected by 2 experienced operators. Qualisys Track Manager software (QTM, Qualisys, Goteborg, Sweden) was used for data recording, processing of trajectory labeling, and data export to c3d files. Time series for sagittal joint angles of the hip, knee, and ankle were then calculated in MATLAB (R2021a, The MathWorks, Inc., Natick, MA, USA) and used for further analysis. All kinematic data were cut for one gait cycle and temporally normalized to 101 data points. Assembled data are from the affected side for patients and the right side for healthy adults.

### 2.3. Kinematic Knee Parameters

**Figure 1:**
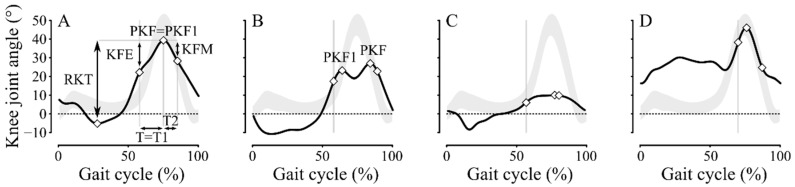
Sagittal knee joint angles for a complete gait cycle in 4 patients (black lines) and mean ± standard deviation (SD) interval in healthy group (grey areas). (**A**) Patient with UKG from *k1* cluster. (**B**) Patient with BKG from *k2* cluster. (**C**) Patient with FLG from *k3* cluster. (**D**) Patient with non-SKG pattern from *k4* cluster. The 5 kinematic parameters used to feed the machine learning algorithm were: (1) the first peak knee flexion angle (PKF1), (2) the angle difference (KFE) between PKF1 and the knee flexion angle at homolateral toe-off (grey vertical bars), (3) the duration from homolateral toe-off to PKF1 (T1), (4) the angle difference (KFM) between PKF1 and the knee flexion angle at the end of the mid-swing when the homolateral tibia is vertical, and (5) the duration from PKF1 to the end of the mid-swing (T2). Total range of knee flexion (RKT) is also shown.

Five selected sagittal kinematic knee parameters that characterize the swing phase and follow standard kinematic definitions [9,11] were collected (Figure 1): (1) the peak knee flexion angle (PKF1); (2) the difference between PKF1 and the knee flexion angle at homolateral toe-off (KFE); (3) the duration between homolateral toe-off and PKF1 (T1, % gait cycle); (4) the difference between PKF1 and the knee flexion angle at the end of the mid-swing when the homolateral tibia is vertical (KFM, Figure 1A); and (5) the duration from PKF1 to the end of the mid-swing (T2, % gait cycle). Note that for PKF1, whenever several bumps were present (e.g., Figure 1B), we only considered the first bump peak angle.

### 2.4. Clustering Procedure, Construct Validity of Classification, and Statistical Analyses

Clusters were determined in MATLAB software (R2021a, The MathWorks, Inc., Natick, MA, USA) using k-means cluster analysis [43,44] applied to these 5 kinematic parameters. This is an iterative method that consists of partitioning a set of n observations into k ≥ 2 clusters defined by centroids, and can be formulated as an optimization problem [45]. A centroid corresponds to the arithmetic mean of all observations belonging to a cluster. The squared Euclidean distance of the observations to the cluster centroids is used as the distance metric. The final clustering solution is the one with the lowest total sum of distances over all replicates. The optimal number of clusters (k) was estimated with the gap statistic [46] using the squared Euclidean distance. A maximum value of 6 was set for k (k ≤ 6) to remain within reasonable clinical use. The optimization of the clusters was performed in MATLAB using the ‘Replicates’ parameter (n = 5), i.e., the number of times to repeat the clustering, each with a new set of initial centroids.

To determine the construct validity of the proposed SKG clusters, we compared it with two existing SKG detection criteria. First, each patient was classified into 3 groups by an experienced operator (F.C.) using the chronic hemiparesis gait classification (CHGC) [47]: group I (*GI*) included patients with reduced ankle dorsiflexion during the swing phase (i.e., equinus) as a kinematic abnormality, group II (*GII*) with SKG, often associated with equinus foot, and group III (*GIII*) with mainly reduced hip excursion in the sagittal plane, often associated with altered kinematics in the other two joints (SKG and equinus). Each group was divided into 2 subgroups depending on whether a knee recurvatum was present or not, with the respective notation a or b. Healthy subjects were grouped as group 0 (*G0*). Second, four gait parameters were calculated for all patients to provide evidence for SKG as presented by Goldberg et al. [9] (see Figure 1B,C: peak knee flexion (PKF), the duration between homolateral toe-off and PKF (T), range of knee flexion from homolateral toe-off to PKF (KFE), total range of knee flexion (RKT)). A knee stiffness score or Goldberg index classified the gait pattern as stiff (score ≥ 3), borderline (score = 2), or not-stiff (score ≤ 1) [9]. For the comparison between the proposed clusters and the CHGC, our hypothesis was that the more severe SKG clusters would only contain the more severely impaired patients (*GII* and *GIII*), and for the comparison with Goldberg index, that, in the more severe SKG clusters, patients will obtain scores ≥ 3. In addition, 20 common sagittal kinematic parameters of the hip, knee, and ankle joints [32,42] (Table 2), as well as 3 clinical measurements and the walking velocity were calculated for the different clusters. Kinematic parameters and walking velocity differences between the proposed clusters were determined by one-way analysis of variance (ANOVA), followed by post hoc Holm-Sidak method for pairwise multiple comparisons. Clinical characteristic (mAS_4ceps_, MRC_hip_, and MRC_ankle_) differences between the proposed clusters were determined by Kruskal–Wallis test, followed by post hoc Dunn method for pairwise multiple comparisons. Kinematic parameters, walking velocity, and clinical characteristic comparisons were performed to confirm the construct validity of the proposed clusters. All statistical analyses were performed in MATLAB.

## 3. Results

**Figure 2:**
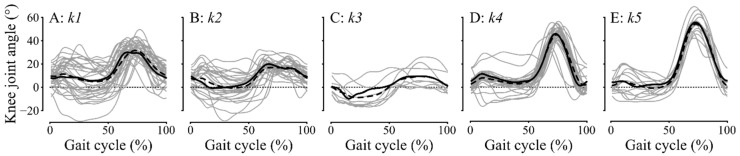
Sagittal knee joint angle for a complete gait cycle. Subplots are: (**A**) *k*1 or UKG, (**B**) *k*2 or BKG, (**C**) *k*3 or FLG, (**D**) *k4* or healthy, (**E**) *k*5 or non-SKG. Individual kinematics are plotted in grey lines, mean kinematics for each cluster are in black dashed lines, and medoids are in continuous black lines.

The optimal number of clusters *k* was five (Figure 2). Two clusters showed a knee pattern in the range of normality or above (*k4* and *k5*), while the other three clusters showed SKG patterns (*k1* to *k3*). In these three clusters, many patients (33/72 or 46%) exhibited a visible discontinuity in the knee flexion curve. Patients in *k1* presented a lower and either delayed knee flexion or a flattened curve (12/34 or 35%), while patients in *k2* mainly showed knee kinematics with double bumps (19/28 or 68%), and patients in *k3* had almost non-existent knee flexion. Therefore, the clusters were named as follows: *k1*: unbend-knee gait (UKG) or mild SKG, *k2*: braked-knee gait (BKG) or moderate SKG, *k3*: frozen-limb gait (FLG) or severe SKG, *k4*: healthy and *k5*: non-SKG. *k5* consisted mainly of post-stroke patients without SKG (14/15 or 93%), *k4* consisted mainly of healthy subjects and a few post-stroke patients (11/28 or 39%), *k1* consisted of post-stroke patients except one healthy subject (1/34 or 3%), *k2* consisted only of post-stroke patients (28/28 or 100%) as well as *k3* (9/9 or 100%). No significant differences were found between the clusters for age, weight, and height.

Our SKG study sample constituted patients with a mean age of 55 years in the chronic stage of stroke disease, with 39% being women, and 45% of patients having a hemiparesis on the left side. The majority of the patients had no increased stretch reflex activity of the *quadriceps femoris* muscle, with a null median value of mAS_4ceps_, a moderate strength decrease in the hip flexor muscles (median value of MRC_hip_: 3), and a severe strength decrease in the ankle plantar flexor muscles (median value of MRC_ankle_: 1).

Clustering results were compared with the CHGC and the Goldberg index (Table 3). A total of 30 out of 115 gait patterns were misclassified (26.1% of errors) between the Goldberg index and the clusters (Table 3: *k1-k2-k3* while the index was *≤*2). Only 7 were misclassified (6.1% of errors) between the CHGC and the clusters (Table 3: *k1* but *G0* and *GI* and *k4*-*k5* but *GII* or *GIII*). Thus, *k4* and *k5* included mainly hemiparetic adults classified as *GI* and healthy subjects, whereas the other three SKG groups included mainly *GII* and *GIII* patients. Moreover, the more severe clusters, *k2* and *k3*, only contained *GII* and *GIII* patients and had a higher median Goldberg index (score = 3) compared to *k1* (score = 2). In all, the comparisons between the clusters and the CHGC or Goldberg index results provided proof of construct validity of the proposed SKG classification.

The clustering results, clinical and gait characteristics, and sagittal kinematic parameters for the five clusters are shown in Table 4. The one-way ANOVA showed a significant difference between the clusters in gait velocity, in all selected knee kinematic parameters defining the clusters, and also in other usual kinematic parameters except H4 and A4. Moreover, post hoc analysis showed several significant differences in adjacent clusters in other usual knee kinematic parameters except K2 and K3.

**Figure 3:**
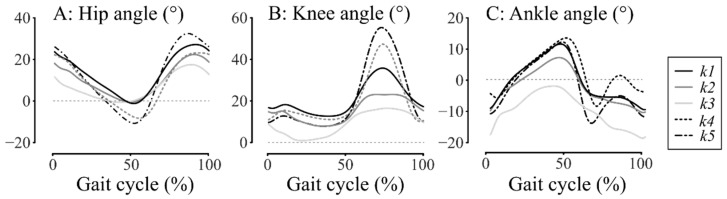
Mean sagittal kinematics of: (**A**) hip joint, (**B**) knee joint, and (**C**) ankle joint. *k1* is the black line, *k2* is the mild grey line, *k3* is the light grey line, *k4* is the black dashed line, and *k5* is the dash-dotted black line.

Interestingly, the post hoc analysis also showed significant differences between adjacent clusters in hip kinematics (H5: *k4* versus *k5* and H6: *k4* versus *k5*, *k1* versus *k2*) and also in ankle kinematics (A3: *k1* versus *k2*, *k2* versus *k3*). Note that ankle strength was also significantly different in adjacent clusters (MRC_ankle_: *k1* versus *k2*). Thus, if there is indeed a gradation of SKG severity from *k1* to *k3* (Figure 3A), hip and ankle joint excursion also appears to be gradually more affected (Figure 3B,C).

## 4. Discussion

The purpose of this study was to develop a new classification system for unilateral SKG kinematic severity using an unsupervised cluster analysis. Our sample of 96 hemiparetic patients with and without SKG and 19 healthy subjects was divided into five clusters. Two clusters included patients without SKG and all healthy subjects. The three remaining SKG clusters were defined according to a three-level severity classification: UKG (mild), BKG (moderate), and FLG (severe). Furthermore, the proposed SKG classification successfully passed the preliminary test for construct validity based on the dual comparison with CHGC and Goldberg score results. 

Of the 96 post-stroke patients analyzed, 72 (75%) had an SKG including 34 (35%) UKG, 28 (29%) BKG, and 9 (9%) FLG. The overall percentage of SKG in our study appears to be close to Kramers De Quervain et al. [7]’s, in which 66% (12/18) of patients had a slow gait velocity and SKG pattern. Before going further in the discussion, it is appropriate to discuss about the external validity of our classification. The external validity is of major clinical importance since it refers to the extent to which our observed SKG clusters established from a monocentric clinical gait analysis database, i.e., a study sample of the target population (patients to whom the study results are intended to be applied in real-world patient populations [37]), is an unbiased estimator of the full target population.

The external validity can be assessed with the generalizability concept that is concerned with making inference from a possibly biased study sample back to the target population [48]. Here, since until today there was no accurate metric definition and diagnostic process of unilateral SKG after stroke, the identification of the target population is far from straightforward, maybe explaining the absence of large-scale, national, and international data sets. To assess the a posteriori generalizability beyond our sample, we can, nevertheless, estimate the differences in the main demographic (sex, age, and weight), clinical (hemiparetic side, time since stroke, mAS_4ceps_, MRC_hip_, and MRC_ankle_), and gait (velocity and K5) variables of our patients compared to those included in previous SKG studies (Table 5). To keep it as concise as possible, our study sample was compared only to the two studies with the largest samples that reported the information. To compare the results as objectively as possible, two different scores were computed: a similarity score (S_score_) between the present and target studies for each variable and a generalizability score (G_score_) between the present and target studies for a same demographic, clinical, or gait characteristic (Table 5). The S_score_, expressed in percent, is computed as the ratio of the values of the two studies (our sample and target population), with always the smaller value as the numerator [49]. If one value is 0, then the score is 0%, and if the two numerator and denominator values are 0, then the score is 100% [49]. The G_score_ is the mean of the S_scores_ for each characteristic [49], here: demographic, clinical, and gait (Table 5). In all, the G_scores_ allow to conclude that our study sample is strongly comparable to other SKG samples for demographic (90%) and gait (95%) characteristics (Table 5). For these latter characteristics, it could be, therefore, considered as almost similar to studies with a more or less comparable sample size. The result was, however, less strong for clinical characteristics (72%, Table 5). This was mainly explained by differences in mAS_4ceps_ and MRC_ankle_ results. However, these studies [22,50,51] included very small sample sizes (13 to 21 SKG patients) and the study [51] only included patients with mAS_4ceps_ ≥ 1+, which could explain the higher median value observed for this clinical parameter (3 versus 1, Table 5). In our study, mAS_4ceps_ that is frequently called Duncan-Ely or Ely test in clinical routine, was assessed but not used as an including criteria since it was shown that this test is not predictive of abnormal electrical activity of the *rectus femoris* muscle during gait in post-stroke SKG patients [52].

An unsupervised k-means method was chosen to develop our SKG classification because we wanted to cluster unlabeled data points to discover hidden patterns in the kinematic knee data without requiring human intervention, i.e., without supervision. The use of semi-supervised or supervised methods was not considered because labelling of the data was not possible here. Indeed, there is no gold standard to classify the severity of SKG. From *k1* to *k3*, the mean values of PKF1 were: 36°, 21°, and 10°, respectively, i.e., a difference of at least 10° between each SKG cluster. According to a recent study [54], the minimal clinically important difference for the range of motion of knee flexion on the affected side during gait in chronic stroke is less than 7°, which is a smaller value than the mean change we observed between our different SKG severity clusters, leading us to conclude that there are three true severity clusters with clinical significance.

Estimation of 99% confidence intervals for maximum knee flexion in swing phase (K5) yielded the following ranges: 33–38° for UKG, 19–24° for BKG, 3–18° for FLG, and 44–50° for healthy subjects. In some previous clinical studies [4,8,18], SKG patients were studied with a maximum knee flexion in the swing phase of up to 45°, a cut-off thus corresponding to our healthy subjects. Therefore, the conclusions of these studies should be taken with caution. To address this issue, we proposed to define SKG for maximum knee flexion in swing phase values strictly below 40°. This cut-off, already used by some authors [20], should be respected in future clinical trials that include SKG patients.

The greatest strengths of our study are that: (1) SKG kinematic severity clusters were derived mathematically from a clinical gait analysis database rather than arbitrarily defined, and (2) it included a large number of patients with SKG (72 patients) compared to previous studies including no more than 47 patients among which non-SKG disorders [7,34]. For these two reasons, we are, therefore, confident that the various cut-off points proposed to define SKG and determine the different severity levels are sufficiently accurate for immediate use by clinicians and researchers.

In addition to the comparison of severity clusters with CHGC and Goldberg score results, one-way ANOVA results also confirmed the construct validity of our classification, which revealed statistically significant differences between clusters for knee kinematic parameters in swing, especially in flexion at toe-off (K4) and peak flexion (K5). Total excursion in the sagittal plane (K6) and mean flexion velocity in the preswing phase (KFV) showed statistical differences, except between clusters *k2* and *k3*, but this could be probably explained by the small sample in *k3*. Furthermore, the proposed classification was supported by its adequacy (i.e., error rate of 6%) with the CHGC [47]. The Goldberg index was basically defined in cerebral palsy but, although no study has assessed its relevance in other populations, is commonly used in adults with post-stroke hemiparesis. However, the comparison between our classification results and the Goldberg index showed a higher error rate (26%) compared to the CHGC. This result can be explained by the fact that this index may present three main problems. Firstly, patients with a score of two out of four are considered borderline (20/115 or 17%), leading to some uncertainty in the diagnosis of SKG. Secondly, the total range of knee flexion (RKT, Figure 1A) included in its assessment can be artificially increased by the presence of a knee recurvatum in the stance phase. This artificial increase may normalize this parameter and, thus, underestimate the presence of SKG. Thirdly, the Goldberg index is only based on knee kinematic parameters and does not take into account the shape of the kinematic curve in the swing phase. In either flattened (mainly in *k1* cluster) or double-bump knee curves (mainly in *k2* cluster), the discontinuity observed in the slope of knee flexion is likely due to a rough braking action of the *quadriceps femoris* muscle that induces an extensor moment. Indeed, some authors [13,14] showed that the knee extensor moment induced by the *quadriceps femoris* muscle, especially the *rectus femoris* head, contributes to the SKG by a decrease in KFV, mainly, when this muscle acts before toe-off. A second possible origin of the SKG is an inadequate ankle push-off. It may result from a decrease in the ankle moment generated by the plantar flexor muscles [55] or in the peak of vertical acceleration of the lateral malleolus [8]. Finally, a third possible origin to SKG is the weakness of the hip flexor muscles [14,56]. In our results, the decrease in KFV was confirmed, as well as a decrease in mean ankle plantarflexion velocity in the preswing phase (MAVP). Total hip excursion in the sagittal plane (H6) also showed some differences between clusters. Therefore, this confirmed that there are multiple mechanisms causing the reduction of peak knee flexion [6] which interact in varying proportions in each cluster leading to increased severity of SKG.

The main therapeutic options for SKG are nerve or muscle surgery or chemo-denervation of selective heads of the *quadriceps femoris* muscle, particularly the *rectus femoris*. Surgery usually consists of selective peripheral neurotomy [17] or distal *rectus femoris* muscle tenotomy [3]. Denervation can be performed by Botulinum toxin [16,21] or phenol injections [12]. Although treatment of *quadriceps femoris* overactivity is required to improve SKG in many cases, the mixed results observed after chemo-denervation [23] are probably due to the difficulty in finding and weighting the contribution of the other causes of SKG. In hemiparetic post-stroke patients with SKG, a higher coactivation of the *quadriceps femoris*–hamstring muscles was observed on the paretic side [57]. The pathophysiological mechanism appears to be extremely complex and could involve other muscles to a greater extent. For example, *vastus intermedius* muscle overactivity is common in patients with SKG and the use of fine-wire electrodes inserted under ultrasound guidance should, therefore, be included in gait analysis to aid clinical decision-making [3,4,5].

Our study has three main limitations that need to be considered. Firstly, only sagittal plane knee kinematic parameters in the swing phase of the gait were used to feed the machine learning algorithm. The inclusion of other knee parameters could be considered, or even other joint movements in other planes, such as hip circumduction [19], to investigate a possible abnormal coordination pattern between the hip and knee. Secondly, additional analysis such as electromyographic patterns should be carried out to better point to the differences between clusters. Thirdly, further research should be conducted to confirm the construct validity of our new classification using a completely different sample of participants.

In an era of patient-customized medicine, machine learning methods enable the determination of specific features to a given individual’s gait pattern and could help clinicians and researchers individualize their analyses, diagnoses, and treatments [58]. Our findings support this and contribute to refining the definition and diagnostic process for SKG in hemiparetic post-stroke patients and will, therefore, provide substantial benefits to the real-world patient population. Three SKG clusters were formed, providing information on the existence of different severity levels with clinical significance, likely due to multifactorial causes.

## 5. Conclusions

This new classification for unilateral SKG, which is a useful complement to the previously developed CHGC based exclusively on visual and clinical examination [47], should be widely adopted by clinicians and researchers to facilitate the identification of factors responsible for SKG and to find the best treatment options for each patient. We also hope that our classification will be an incentive to collect national and international data sets on SKG that are sorely lacking to date.

## Figures and Tables

**Table 1:** Demographic, clinical, and gait characteristics of the participants (all patients and healthy subjects). Values are expressed in numbers, mean ± SD, or median and [1st quartile–3rd quartile].

Characteristics	All Patients (n = 96)	Healthy (n = 19)
**Demographic**		
Sex (w/m) Age (years) Weight (kg) Height (m)	41/55 53 ± 12 79 ± 17 1.69 ± 0.08	10/9 54 ± 7 73 ± 14 1.74 ± 0.07
**Clinical**		
Hemiparetic side (l/r) Time since stroke (months) mAS_4ceps_ (0–4) MRC_hip_ (0–5) MRC_ankle_ (0–5)	49/47 49 ± 69 0 [0–1] 4 [3–4] 2 [1–3]	NA NA NA NA NA
**Gait**		
Velocity (m s^−1^)	0.64 ± 0.28	0.59 ± 0.07
K5 (°)	26 ± 9	23 ± 7

w: women, m: men, l: left, r: right, mAS_4ceps_: modified Ashworth scale for *quadriceps femoris* muscle, MRC_hip_: hip flexor muscles’ strength according to Medical Research Council, MRC_ankle_: ankle plantar flexor muscles’ strength according to Medical Research Council, K5: maximum knee flexion in swing phase, NA: not applicable.

**Table 2:** Description of the kinematic parameters computed for the hip, knee, and ankle joints.

Joints	Abbreviation	Description	Units	References
**Hip**				
	H1	Hip joint angle at initial contact	°	[32,47]
	H2	Maximum hip flexion during loading phase	°	[32,47]
	H3	Maximum hip extension in stance phase	°	[32,47]
	H4	Hip joint angle at toe-off	°	[32,47]
	H5	Maximum hip flexion in swing phase	°	[32,47]
	H6	Total hip excursion in sagittal plane	°	[32,47]
**Knee**				
	K1	Knee joint angle at initial contact	°	[32,47]
	K2	Maximum knee flexion during loading phase	°	[32,47]
	K3	Maximum knee extension in stance phase	°	[32,47]
	K4	Knee joint angle at toe-off	°	[32,47]
	K5	Maximum knee flexion in swing phase	°	[32,47]
	K6	Total knee excursion in sagittal plane	°	[32,47]
	KFV	Mean knee flexion velocity in preswing phase	° s^−1^	[8]
**Ankle**				
	A1	Ankle joint angle at initial contact	°	[32,47]
	A2	Maximum ankle plantarflexion during loading phase	°	[32,47]
	A3	Maximum ankle dorsiflexion in stance phase	°	[32,47]
	A4	Ankle joint angle at toe-off	°	[32,47]
	A5	Maximum ankle dorsiflexion in swing phase	°	[32,47]
	A6	Total ankle excursion in sagittal plane	°	[32,47]
	A7	Maximum ankle plantarflexion in swing phase	°	[32,47]
	MAVP	Mean ankle plantarflexion velocity in preswing phase	° s^−1^	-

**Table 3:** Comparison of SKG classification following Goldberg index (i.e., SKG if score > 2) and CHGC (i.e., SKG for groups *GII* and *GIII*) versus clustering (i.e., SKG for *k1* to *k3*). Misclassifications are highlighted in grey and overall error rates are given for both methods.

	*k1*	*k2*	*k3*	*k4*	*k5*	Error/Total (%)
	UKG (Mild)	BKG (Moderate)	FLG (Severe)	Healthy	Non-SKG	
**Goldberg index**						30/115 (26.1)
0	1	0	0	17	1	
1	9	0	0	7	14	
2	13	5	2	4	0	
3	11	12	5	0	0	
4	1	11	2	0	0	
**CHGC**						7/115 (6.1)
*G0*	1	0	0	17	1	
*GIa*	1	0	0	4	4	
*GIb*	0	0	0	3	9	
*GIIa*	16	9	0	1	1	
*GIIb*	15	13	4	2	0	
*GIIIa*	1	2	1	0	0	
*GIIIb*	1	4	4	1	0	

UKG: unbend-knee gait, BKG: braked-knee gait, FLG: frozen-limb gait.

**Table 4:** Clustering results, clinical and gait characteristics (median and [1st–3rd quartiles]), and sagittal kinematic parameters (mean ± SD) for the different clusters (*k1* to *k5*) and results of the one-way ANOVA or Kruskal–Wallis tests.

	*k5*	*k4*	*k1*	*k2*	*k3*	*p*
	Non-SKG	Healthy	UKG (Mild)	BKG (Moderate)	FLG (Severe)	
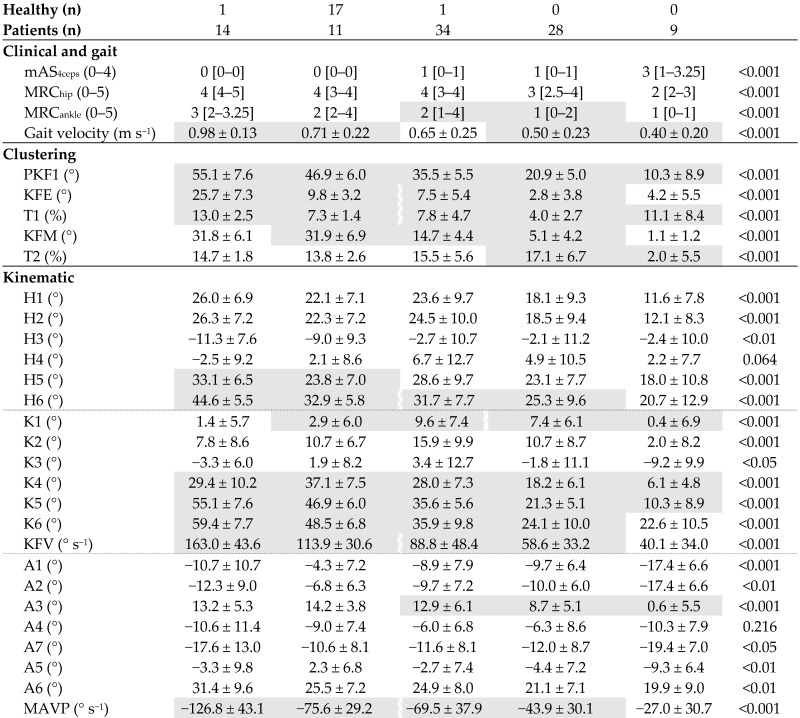						

UKG: unbend-knee gait, BKG: braked-knee gait, FLG: frozen-limb gait, mAS_4ceps_: modified Ashworth scale for *quadriceps femoris* muscle, MRC_hip_: hip flexor muscles’ strength, MRC_ankle_: ankle plantar flexor muscles’ strength. Post hoc significant differences between adjacent clusters are in continuous grey and a vertical broken line indicates that this is not the case between these two adjacent clusters. Description of all abbreviations for kinematic parameters are shown in Table 2.

**Table 5:** Comparison between the present SKG sample and other target samples from the literature using S_scores_ and G_scores_. Values are expressed in numbers, mean ± SD, or median and [interquartile range] for samples and in percent for S_scores_ and G_scores_.

	Present SKG Sample	Target Sample(s) [Reference]	S_score_	G_score_
**Demographic**				90
Sex (% women)	39	45 [18] 30 [52]	87 77	
Age (years)	55 ± 11	55 ± 14 [18] 57 ± 13 [52]	100 96	
Weight (kg)	80 ± 16	74 ± 12 [18] 67 ± 11/73 ± 8 [22]	93 84/91	
**Clinical**				72
Hemiparetic side (% left)	45	31 [18] 48 [52]	69 94	
Time since stroke (months)	54 ± 75	83 ± 71 [52] 53 ± 49 [20]	65 98	
mAS_4ceps_	1 [1]	2 [1]/2 [2,22] 1 [1]/2.5 [1,50]	50 100/40	
MRC_hip_	3 [1]	3 [3,17]	100	
MRC_ankle_	1 [2]	3 [2,51]	33	
**Gait**				95
Velocity (m s^−1^)	0.56 ± 0.25	0.58 ± 0.25 [53] 0.57 ± 0.20/0.54 ± 0.18 [22]	97 98/96	
K5 (°)	25 ± 10	25 ± 9 [8] 30 ± 12 [18]	100 83	

mAS_4ceps_: modified Ashworth scale for quadriceps femoris muscle, MRC_hip_: hip flexor muscles’ strength according to Medical Research Council, MRC_ankle_: ankle plantar flexor muscles’ strength according to Medical Research Council, K5: maximum knee flexion in swing phase.

## Data Availability

The data presented in this study are not available on request from the corresponding author and not publicly available since, although de-identified, no special data-sharing consent was retrospectively obtained from the participants.
